# Vascular and Circulating Blood Exposure to Proton or Photon Irradiation: Differentials in Lymphocyte Subset Metrics in Cancer Patients

**DOI:** 10.3390/cancers18213587

**Published:** 2026-11-08

**Authors:** Rosa Meiriño, Felipe A. Calvo, Enrique Amaya, Javier Burguete, Javier Serrano, Marina García Cardosa, Diego Pedrero, Diego Azcona, Jacobo Palma, María A. Fortuño, Mauricio Cambeiro, Marta Lasa, Elena Antolín San Martín, Marta Vidorreta, Javier Aristu

**Affiliations:** 1 Department of Radiation Oncology, Clínica Universidad de Navarra, 28027 Madrid, Spain; rmeirino@unav.es (R.M.); eamayae@unav.es (E.A.); fserranoa@unav.es (J.S.); dpedrerodea@unav.es (D.P.); jazcona@unav.es (D.A.); jpalmadelga@unav.es (J.P.); mcambeiro@unav.es (M.C.); eantolinsan@unav.es (E.A.S.M.); jjaristu@unav.es (J.A.); 2 Department of Physics and Applied Mathematics, Universidad de Navarra, 31008 Pamplona, Spain; javier@unav.es (J.B.); mgarciacard@unav.es (M.G.C.); 3 Biobank, Centro de Investigación Médica Aplicada (CIMA), Universidad de Navarra, 31008 Pamplona, Spain; fortuto@unav.es; 4 Centro de Investigación Médica Aplicada (CIMA), Universidad de Navarra, 31008 Pamplona, Spain; mlasav@unav.es; 5 Siemens Healthineers, 28033 Madrid, Spain; marta.vidorreta@siemens-healthineers.com

**Keywords:** radiation-induced lymphopenia, effective dose to immune cells, proton therapy, vascular dosimetry, integral dose, phase-contrast MRI, vessel segmentation, immunophenotyping, organs at risk, immune-sparing radiotherapy, radiation blood exposure

## Abstract

Background/Objectives: Radiation-induced lymphopenia is consistently associated with adverse outcomes across solid tumours, yet the vascular compartment that carries the irradiated lymphocyte pool is not routinely delineated during treatment planning. The VASA project makes blood vessels an explicit, measurable structure in that process. Phase II aimed primarily to establish the feasibility of that delineation and secondarily to explore how dose–volume metrics computed on MRI-segmented vessels relate to serial immunophenotyping in patients treated with protons or photons. Methods: Thirty-nine patients treated between March 2021 and May 2022 were studied prospectively (30 proton, 9 photon). Flow-contrast three-dimensional MRI with three velocity-encoding directions was added to simulation; vessel masks segmented semi-automatically from the sum-of-magnitude reconstruction were imported into the treatment-planning system to generate dose–volume histograms. The effective dose to circulating immune cells (EDIC), the integral dose to vessels (IDVe) and the integral dose to target were computed. Blood was sampled at the start and at the end of irradiation for lymphocyte counts and B, T and NK immunophenotyping. Groups were compared with Welch’s *t*-test for continuous variables and Fisher’s exact test for categorical variables, without correction for multiple comparisons; the photon subgroup was not matched. Results: EDIC was higher with photons than protons (5.60 vs. 1.25 Gy; *p* = 0.005) and in patients who became lymphopenic (4.84 vs. 1.47 Gy; *p* = 0.027); IDVe differed in the same direction without reaching significance (3.60 vs. 0.53 Gy·L, *p* = 0.091; 3.08 vs. 0.67 Gy·L, *p* = 0.180). Within the proton cohort, EDIC and IDVe increased from brain to head-and-neck to thorax–abdomen targets (0.81, 1.11 and 2.17 Gy; 0.13, 0.26 and 1.63 Gy·L), and the IDVe ordering persisted after adjustment for target volume (*p* < 0.001). Lymphocyte counts fell from 1.92 to 1.43 × 10^3^/µL in the proton cohort (*p* < 0.001), with the lowest end-of-treatment values in thorax–abdomen targets (1.31 × 10^3^/µL). B, T and NK subpopulations all declined in the proton cohort; among regions, only head-and-neck targets showed significant T and NK losses. Conclusions: Patient-specific vascular delineation and dosimetry are feasible in a clinical planning workflow, and the resulting metrics track irradiation modality and the magnitude of lymphocyte depletion. These associations are exploratory; immune-sparing plan optimisation using vascular constraints remains a future application requiring prospective validation.

## Simple Summary

Radiotherapy is given to more than half of all patients with cancer. While the radiation beam is aimed at the tumour, blood keeps flowing through the treated region, so the immune cells carried in circulation are irradiated as well. Patients whose lymphocyte counts decline during treatment tend to have worse outcomes, yet blood vessels are not contoured, measured or spared during treatment planning. Here we mapped the vascular tree of 39 patients with a magnetic resonance sequence that makes flowing blood visible, added it to the radiotherapy plan as an organ to protect, and measured its dose. Patients treated with protons received a much lower dose to their vessels and circulating blood than those treated with photons, and lost fewer lymphocytes. The study shows the measurement is feasible in routine practice; the link between vascular dose and immune depletion is an association that larger studies must confirm.

## 1. Introduction

“Radiotherapy centers per population” is the only therapeutic variable that significantly results in less cancer death as reported in a machine learning methodology analysis recently published that evaluated cancer statistics in 185 countries [1]. Its therapeutic index has historically been defined by the balance between tumour control and late damage to the solid organs that lie within or adjacent to the irradiated volume. Over the last decade a third compartment has forced its way into that equation: the immune system. Peripheral lymphocytes are among the most radiosensitive cells in the human body, undergoing interphase death after fractional doses well below 1 Gy—a fragility recognised in the clinic within a few years of the first fractionated courses [2]—and they are the only cell population that is irradiated in every patient regardless of where the beam is pointed, because they are continuously transported through the treated volume by the circulation [3,4,5]. That radiosensitivity is not uniform across individuals: systematic dissection of the acute and persistent immune response to irradiation in a genetically diverse murine population has shown that the magnitude and persistence of lymphocyte depletion are themselves heritable traits mapping to discrete loci [6], and in patients the in vitro radiosensitivity of peripheral lymphocytes and its transcriptional correlates track the severity of late normal-tissue reactions [7]. Genetic background is therefore expected to modulate how any given blood dose translates into measured lymphopenia. The consequence is a systemic, dose-dependent depletion of circulating lymphocytes that is neither predicted by conventional organ-at-risk (OAR) dose–volume constraints nor recorded by the dose–volume histograms (DVHs) generated during routine planning [8].

The clinical weight of this phenomenon is now well established. A systematic review and meta-analysis of radiation-induced lymphopenia (RIL) across solid tumours found a consistent and detrimental prognostic association between severe lymphopenia and overall survival (OS) [9], confirming earlier syntheses of the same literature [10] and the pooled analysis of four independent solid-tumour cohorts in which treatment-related lymphopenia below 500 cells/mm^3^ identified patients with reduced survival [11]. The association has since been reproduced, with broadly comparable effect sizes, in thoracic tumours [12], head and neck cancer [13], pancreatic adenocarcinoma [14,15], glioblastoma [16,17] and oesophageal cancer [18], and formal mediation analysis in oesophageal cancer suggests that lymphopenia lies on the causal path between treatment and survival rather than simply marking a frailer patient [19]. Lymphopenia is therefore not only a laboratory abnormality; it is a measurable erosion of the immunocompetence on which both endogenous tumour surveillance and modern immune checkpoint therapy depend [20,21].

The mechanistic account of RIL that has shaped the field originates in the observation that the dose received by circulating cells cannot be interpreted as a static DVH. Modelling a conventional glioblastoma plan, Yovino and colleagues estimated that the overwhelming majority of the circulating blood pool receives a low but biologically meaningful dose over a fractionated course, which was sufficient to explain the lymphopenia observed clinically [22]. That insight was formalised as the effective dose to circulating immune cells (EDIC), an equivalent uniform dose to the whole blood compartment reconstructed from the doses to blood-containing organs, blood flow and the number of fractions [23]. In the secondary analysis of NRG/RTOG 0617, EDIC behaved as an independent predictor of tumour control and overall survival in stage III non-small-cell lung cancer [24], and the metric has since been associated with lymphocyte nadir and survival in oesophageal cancer [25], limited-stage small-cell lung cancer [26], breast cancer [27] and triple-negative breast cancer treated with helical tomotherapy [28].

A second generation of dosimetric tools has moved beyond the static EDIC approximation towards explicit simulation of blood transport. Four-dimensional blood-flow models track individual blood particles through a time-resolved dose distribution [29], frameworks such as HEDOS couple whole-body blood-flow simulations to the delivered plan [30], stochastic [31] and organ-specific [32] implementations have followed, and dedicated packages now estimate lymphocyte dose for brain radiotherapy [33]. These models have already produced clinically actionable predictions—prolonged fraction delivery times increase the dose absorbed by circulating lymphocytes [34], and dynamic blood dose correlates with clinical outcome in head-and-neck cancer [35]—and the field has been critically reviewed [36]. Their common limitation is anatomical: blood transport is simulated on reference or population-average vasculature, and inter-subject cerebrovascular variability alone has been shown to be a source of uncertainty in the computed dose to circulating cells [37]. The patient’s own vascular tree is missing from the calculation because it is not imaged, not segmented and not contoured. Recent papers have included the role of patient-specific blood circulation, but have not considered the involved biological effects [38,39].

Particle therapy as an immune-sparing modality is an available alternative to photontherapy. Protons minimise exit dose in the irradiated volume, which decreases the fraction of the circulating blood pool that passes through a non-negligible dose region during each fraction, a substantial effect when compare to photons for an equivalent target. Propensity-matched and comparative series in oesophageal cancer have repeatedly shown lower rates of severe lymphopenia with proton beam therapy than with intensity-modulated radiotherapy [40,41,42], a finding extended by the comprehensive analysis of a randomised phase IIB trial [43], and reduced haematologic toxicity has been documented in medulloblastoma treated with proton craniospinal irradiation [44]. EDIC itself is lower with protons in mediastinal Hodgkin lymphoma [45] and breast cancer [46], carbon ions further reduce the risk of severe lymphopenia in locally advanced pancreatic cancer [47], and modality- and technique-dependent immune sparing is now being modelled prospectively [48,49] and tested in dedicated translational protocols [50]. The recent randomised comparison of intensity-modulated proton therapy with IMRT in oropharyngeal cancer establishes that modality choice can be interrogated at the level of a phase III endpoint [51].

Vascular structures, meanwhile, have entered the planning process by a different door and for a different reason. Dose–volume histograms have been a standard of normal-tissue evaluation since the 1980s [52], but large vessels were first contoured systematically only around 2010, when carotid-sparing techniques were developed for early glottic cancer and for re-irradiation [53], and the vessel-updated constraints— for the aorta and great vessels in stereotactic regimens—were derived to prevent high-dose late events such as rupture and stenosis [54]. These recommendations do not mention the low-to-intermediate dose bath that determines how much of the circulating lymphocyte pool is irradiated, and the impact over the endothelium, whose radiation injury is itself a recognised mechanism of long-term vascular morbidity [55]. The vascular compartment is therefore simultaneously the conduit of the most radiosensitive cell population in the body and one of the few structures that routine dosimetry does not describe.

What is still absent from this literature is the determination of the biological effect of a patient-specific, image-derived representation of the vascular compartment itself. Systematic reviews of dose constraints for lymphocyte sparing conclude that no standardised constraint exists for the circulating pool [56], and the structures currently proposed as surrogates—spleen [57,58], thymus [59,60], bone marrow [61], major lymphatic volumes [62]—are static organs that contain lymphocytes rather than the conduits through which the whole pool repeatedly passes. The VASA project was designed to close that gap by making blood vessels an explicit, delineated and measurable structure in the planning process. In Phase I, EDIC and integral dose to vessels were computed in 20 patients; those with EDIC > 4.0 Gy experienced more grade 3–4 lymphopenia than those with EDIC ≤ 4.0 Gy, consistent with published series, and the same trend was observed above an integral vessel dose of 1 Gy·L although the cohort was too small to be conclusive.

The present report describes Phase II of the VASA project. Its objectives were:

1. To demonstrate that the vascular tree can be segmented from flow-contrast MRI acquired at simulation and imported into the treatment-planning system as an organ at risk, so that vessel DVHs are produced patient by patient;
2. To quantify the effective dose to circulating immune cells (EDIC), the integral dose to vessels (IDVe) and the integral dose to target (ID-TV) in a prospective cohort treated with protons or photons;
3. To relate these dosimetric assessments to serial lymphocyte counts and to B, T and NK subpopulations measured at two timepoints during the irradiation course; and
4. To establish whether the dosimetric differential between modalities, proton vs. photon, and between anatomical target regions is reflected in the magnitude of lymphocyte depletion.

These objectives correspond to three distinct levels of evidence, which are kept separate throughout this report. The first is technical feasibility—whether a patient-specific vascular tree can be segmented, imported and dosimetrically characterised in a clinical workflow—and it is the level the present design is able to establish. The second is the exploratory association between vascular dose and the magnitude and composition of lymphocyte depletion, which this cohort can describe but, being unmatched and without mature clinical endpoints, cannot establish as causal. The third is the prospective clinical application of vascular sparing, which remains a hypothesis to be tested and is presented as such.

## 2. Materials and Methods

### 2.1. Study Design and Population

This is a prospective, single-institution observational study conducted at the Department of Radiation Oncology, Clínica Universidad de Navarra (Madrid, Spain), as Phase II of the VASA project (VAsos y SAngre, Spanish for vessels and blood), whose design and earlier findings have been described previously [8]. Phase I established the feasibility of deriving vessel dose–volume histograms from CT angiography in patients treated with photons; Phase II, reported here, replaces CT angiography with flow-contrast MRI and adds the comparison with proton therapy. Patients treated with a radiotherapy component between March 2021 and May 2022 were enrolled consecutively. Eligibility required an indication for external beam radiotherapy with curative intent, the ability to undergo an additional MRI acquisition at the time of simulation, and availability for serial peripheral blood sampling during the irradiation course. Thirty-nine patients were analysed, 27 men and 12 women; 30 received proton therapy and 9 received photon therapy. Sex, tumour site and histology, prescription dose and fractionation, clinical target volume, vascular comorbidity, toxic habits, prior and concurrent systemic therapy and serial absolute lymphocyte counts were recorded for every patient and are compared between the two modality groups in Table 1. Stage, treatment intent, prior surgery, the specific delivery technique, gross tumour volume and corticosteroid exposure were not captured by the study database and are accordingly absent from Table 1. The photon subgroup corresponds to a work-in-progress recruitment stream that remains open, and its size is therefore smaller than that of the proton cohort by design rather than by attrition; this asymmetry is carried through the interpretation of every modality comparison reported below.

Within the proton cohort, patients were grouped by the anatomical region containing the irradiation target into brain (*n* = 11), head and neck (*n* = 12) and thorax–abdomen (*n* = 7). The composition of each group, including the distribution of focal versus extended irradiation volumes, is summarised in Table 1. The study workflow, from image acquisition through vascular dosimetry to immunophenotyping, is shown in Figure 1.

### 2.2. Flow-Contrast MR Imaging and Vessel Segmentation

The vasculature and the velocity of flow within it were recovered using a dedicated MR protocol added to the simulation session. Imaging was performed on a 3 T MAGNETOM Vida system (syngo MR XA20A; Siemens Healthineers, Erlangen, Germany). A 3D T1 VIBE Dixon acquisition was obtained first for morphological assessment and co-registration, followed by MR flow imaging. Flow imaging used a three-dimensional phase-contrast (PC) prototype sequence with velocity encoding applied along three orthogonal directions (right–left, anterior–posterior and through-plane), so that the full velocity vector is characterised rather than a single projection of it. For thoracic and abdominal targets the sequence incorporated the ReCAR respiratory navigator, which orders k-space acquisition according to the detected respiratory position and thereby raises the navigator acceptance rate relative to conventional gating. The imaging volume was prescribed to match the coverage of the planning CT, with a craniocaudal extent of approximately 20–22 cm. Spatial resolution was matched to the calibre of the vessels in the target region—1 mm for head acquisitions and 1.7 mm for body acquisitions, adjusted per axis within a 0.75–2 mm range according to vessel size and the dominant direction of flow. The two flow acquisitions together added 10–20 min to the simulation session, depending on the anatomical region, the use of the navigator and the patient’s respiratory rate [8].

Two separate flow acquisitions were performed per patient, with the velocity-encoding parameter (VENC) optimised separately for arterial and for venous flow, because a single VENC cannot simultaneously avoid aliasing in high-velocity arterial segments and preserve signal in slow venous return. VENC values were set to 50 and 20 cm/s for head-and-neck acquisitions and raised for thoracic, abdominal and pelvic acquisitions to accommodate the faster flow in the descending aorta and inferior vena cava (Table 2). For each acquisition, three phase images and one magnitude sum (MSUM) image were reconstructed, the MSUM image being formed from the magnitude images of all three flow-encoding directions; it highlights voxels in which flow is present, and the grey value of a voxel increases with flow velocity. A rephased image acquired without velocity encoding was used for MR-to-CT registration. This flow-weighted contrast is what allows the vascular tree to be recovered without an exogenous contrast agent, and it is the same physical principle exploited by 4D flow MRI in cardiovascular imaging [63,64].

Segmentation was performed semi-automatically on the MSUM image in syngo.via (Siemens Healthineers, Forchheim, Germany). An intensity-based region-growing step generated the initial vessel mask from seed points placed in the principal arterial and venous trunks of the imaged region, with the intensity threshold set on the MSUM histogram of each individual acquisition rather than on a fixed value, because the flow-weighted grey scale is not quantitatively comparable between VENC settings. The resulting mask was then reviewed slice by slice and corrected manually by a single trained radiation oncologist, who removed spurious connections to regions of flow-unrelated hyperintensity and restored vessel segments lost where the velocity component along the encoding direction approached zero; custom scripts were used to enforce anatomical correctness and connectivity of the resulting tree, an approach analogous to the automated and atlas-based vessel-extraction strategies developed for 4D flow MRI [65,66]. All masks in this series were produced by that same observer and a second independent delineation was not performed, so neither interobserver nor intraobserver reproducibility was quantified; this is a recognised limitation, since interobserver variability in organ-at-risk delineation is substantial even for well-established structures [67], and a formal reproducibility study with multiple observers is planned for the next phase of the project. The delineation protocol differed between anatomical regions in VENC, navigator use and the trunks used as seeds, and the practical consequences of those differences are summarised in Figure 2. The procedure recovers the vascular tree from the large arteries and veins down to vessels whose diameter approaches the acquired voxel size, which in practice means a reliable lower bound of roughly two voxels across—about 2 mm for head acquisitions and about 3.5 mm for body acquisitions. Below that calibre the measured signal is a volume-weighted mixture of blood and surrounding tissue, so a vessel either fails to reach the region-growing threshold and is omitted or is included with an overestimated cross-section; partial-volume effects of this kind are the dominant source of error in phase-contrast quantification of small vessels and are not removed by the segmentation step [68]. No partial-volume correction was applied. The mask generated here is therefore a validated representation of the large-vessel compartment only, and not of the entire vascular volume through which lymphocytes circulate: the arteriolar, capillary and venular beds, which carry a substantial share of the blood volume and the whole of the exchange surface, are below the resolution of any clinically feasible acquisition and are not represented. IDVe should accordingly be read as the energy deposited in the conducting vessels that traverse the irradiated region, which is a measurable surrogate for, and not a measurement of, the exposure of the circulating pool. The final vessel mask was exported to the RayStation treatment-planning system (RaySearch Laboratories, Stockholm, Sweden), registered to the planning CT and treated exactly as any other organ at risk, so that a vessel DVH is produced alongside the conventional OAR DVHs [69]. Arterial and venous structures were both retained in the mask, which is what distinguishes this MRI-based workflow from the CT-angiographic approach used in Phase I of the project, in which the venous compartment was poorly resolved. In parallel, the phase images were corrected for aliasing and the three-dimensional velocity fields were reconstructed offline in MATLAB (MathWorks, Natick, MA, USA). These fields were used for an in silico determination of the dose received by the circulating blood [38,39]. They are the substrate for the blood-transport modelling planned for the next phase of the project where we will combine the biological effects and the calculated dose.

### 2.3. Radiotherapy Planning and Dosimetric Endpoints

All patients were planned on the simulation CT with the MRI-derived vessel mask registered to it. Proton treatments were delivered with pencil beam scanning; photon treatments were delivered with intensity-modulated techniques. Dose–volume histograms were generated for the target, for the conventional organs at risk and for the segmented vascular structure. Three dosimetric endpoints were extracted for each patient.

The effective dose to circulating immune cells (EDIC) was calculated according to the published formulation [23,24]:

(1) $$\mathrm{EDIC}\;=\;0.12\;{\bar{D}}_{\mathrm{lung}}\;+\;0.08\;{\bar{D}}_{\mathrm{heart}}\;+\;0.15\times 0.85\;{\bar{D}}_{\mathrm{liver}}{\left(\frac{n}{45}\right)}^{1/2}\;+\;\left[0.45\;+\;0.35\times 0.85{\left(\frac{n}{k}\right)}^{1/2}\right]\frac{\mathrm{ITD}}{{62\times 10}^{3}}$$

where ${\bar{D}}_{\mathrm{lung}}$, ${\bar{D}}_{\mathrm{heart}}$ and ${\bar{D}}_{\mathrm{liver}}$ are the mean lung, mean heart and mean liver doses respectively, ITD is the integral total dose over the scanned body region, *n* is the number of fractions and k is a model constant. The expression reconstructs an equivalent uniform dose to the whole blood compartment from the doses absorbed by the principal blood-containing organs, weighted by their contribution to the circulating pool and corrected for fractionation. The equation has been re-typeset here from the published formulation [23,24] rather than reproduced from it.

The integral dose to vessels (IDVe) and the integral dose to the target volume (ID-TV) were computed as the product of the mean dose and the volume of the corresponding structure, and are expressed in Gy·L:

(2) $$ID=\bar{D}\times V$$

where $\bar{D}$ is the mean dose to the structure and V its volume. IDVe therefore quantifies the total energy deposited in the delineated vascular compartment, and complements EDIC: where EDIC estimates what the moving blood pool absorbs on average, IDVe measures what the conduits that carry it actually receive in the individual patient’s anatomy.

### 2.4. Blood Sampling and Immunophenotyping

Peripheral blood was drawn at two timepoints in every patient: in the first week of the irradiation course (start) and within the last days of treatment (end). The schedule is therefore defined by the fraction of the course elapsed rather than by a fixed number of weeks, so the absolute interval between samples varies with the length of the prescribed course; the elapsed time in weeks at each sampling point is reported in Table 1 by modality group, and the consequences of this design for the interpretation of the lowest recorded value are addressed in Section 3.5 and Section 4.6. Samples were processed by the Biobank of the Centro de Investigación Médica Aplicada (CIMA, University of Navarra). Complete blood counts provided the absolute lymphocyte count (ALC, ×10^3^/µL). Multiparameter flow cytometry was used to quantify the B, T and NK lymphocyte subpopulations, reported as a percentage of leucocytes. For each patient the following immunological endpoints were derived: ALC at start and at end of treatment, the absolute change in ALC between these two timepoints (Δ lymphocytes), the lower of the two recorded ALC values, and the start-to-end change in each subpopulation. Lymphopenia was graded from the ALC according to the Common Terminology Criteria for Adverse Events v5.0 [70]: grade 1, ALC below the lower limit of normal to 0.8 × 10^3^/µL; grade 2, 0.5 to below 0.8 × 10^3^/µL; grade 3, 0.2 to below 0.5 × 10^3^/µL; and grade 4, below 0.2 × 10^3^/µL. Throughout this report a patient is classified as having developed lymphopenia when the lower of the two recorded ALC values fell below 0.8 × 10^3^/µL, that is, CTCAE grade 2 or worse; the grade distribution is given in Table 1. Because subpopulations were quantified as a percentage of leucocytes rather than as absolute concentrations, no CTCAE grading is applicable to them.

### 2.5. Statistical Analysis

Continuous variables are reported as group means with the standard deviation and the 95% confidence interval of the mean; differences between two groups are reported as the difference in means with its 95% confidence interval, so that the precision of each estimate can be judged independently of the significance test. Comparisons of means between two groups, and pairwise comparisons between the three anatomical regions of the proton cohort, were performed with Welch’s *t*-test and exact two-sided *p*-values are given to three decimal places, or as *p* < 0.001 below that resolution, rather than as a threshold statement; a two-sided *p* < 0.05 was considered statistically significant. Categorical clinical and treatment variables were compared between the proton and photon groups with Fisher’s exact test, or with the chi-square test where expected cell counts allowed it, and the test used is stated for every comparison in Table 1. Pairwise significance between anatomical regions is reported in the tables using the superscript notation a (brain), b (head and neck) and c (thorax–abdomen), so that, for example, “*p* < 0.05 a–c” denotes a significant difference between the brain and thorax–abdomen groups. Correlations between dosimetric distributions and lymphocyte endpoints were examined across the whole series and separately within the proton cohort. Given the exploratory nature of this phase of the project and the size of the photon subgroup, no correction for multiple comparisons was applied; with the number of comparisons reported here the familywise error rate is accordingly inflated, and no individual *p*-value should be read as confirmatory evidence. No multivariable model was fitted, because the number of events does not support one. All inferential statements in this report are therefore descriptive and hypothesis-generating, and the feasibility result is the only conclusion the design supports without qualification.

### 2.6. Ethics and Data Statements

The study was conducted in accordance with the Declaration of Helsinki and approved by the Institutional Review Board/Research Ethics Committee of the Universidad de Navarra (protocol code 2021.011 CEI, approval date 11 February 2021). Written informed consent was obtained from all participants before enrolment. Generative artificial intelligence was not used to generate study data, dosimetric calculations or statistical results; any use in the preparation of the manuscript is disclosed in the Acknowledgments.

## 3. Results

### 3.1. Vascular Segmentation and Cohort Dosimetry

The first level of evidence addressed by this study is technical. Flow-contrast MRI, vessel segmentation and import into the treatment-planning system were completed in all 39 of 39 patients in whom they were attempted, with no acquisition aborted for claustrophobia, motion or navigator failure, and a vessel DVH was generated for every plan. How the vascular tree was defined, and how that definition differs between anatomical regions, is shown in Figure 2. The procedure added a single approximately 20 min acquisition to the simulation session and did not require modification of the immobilisation, planning or delivery workflow. For the series as a whole the mean effective dose to circulating immune cells was 2.25 Gy (SD 2.63; 95% CI 1.40–3.10) and the mean integral dose to the segmented vascular tree was 1.18 Gy·L (SD 2.41; 95% CI 0.38–1.97; *n* = 38, one vessel DVH not retrievable). The baseline clinical and treatment characteristics of the proton and photon groups are compared in Table 1; the groups differed in age (66.3 vs. 46.7 years, *p* = 0.013), in the distribution of target regions (*p* = 0.014), in the prevalence of vascular comorbidity (*p* = 0.002) and in baseline ALC (1.30 vs. 1.92 × 10^3^/µL, *p* = 0.012), and did not differ detectably in sex (*p* = 0.228), prescription dose (*p* = 0.161), number of fractions (*p* = 0.248), target volume (*p* = 0.156), previous chemotherapy or radiotherapy (*p* = 1.000), toxic habits (*p* = 1.000) or concurrent systemic treatment (*p* = 0.669), and every subsequent modality comparison is interpreted in the light of those imbalances. The remainder of this section reports the second level of evidence—exploratory associations between vascular dose and immune-cell endpoints—and should be read as such.

### 3.2. Proton Versus Photon Differentials

The two irradiation modalities separated sharply on both metrics. Patients treated with photon RT received a mean EDIC of 5.60 Gy (SD 3.48) compared with 1.25 Gy (SD 1.10) in patients treated with proton RT (difference 4.35 Gy, 95% CI 1.77–6.93; *p* = 0.005), and a mean integral dose to vessels of 3.60 Gy·L (SD 4.43) compared with 0.53 Gy·L (SD 0.79; difference 3.07 Gy·L, 95% CI −0.63 to 6.77; *p* = 0.091) (Figure 3, Table 3). The differential is therefore approximately four-and-a-half-fold for the dose absorbed by the circulating immune compartment and approximately seven-fold for the energy deposited in the vascular tree itself, although with nine photon-treated patients only the EDIC difference reaches conventional significance and the IDVe interval is compatible with no difference. Both photon values lie above, and both proton values below, the corresponding whole-series means, so the series average is an artefact of cohort composition rather than a meaningful descriptor of either modality.

Because the two modality subgroups are not matched, one patient with an upper-abdominal target was replanned in silico for both modalities at the same prescription (40 Gy in 20 fractions), on the same planning CT and with the same vessel masks, so that the dosimetric differential could be inspected with anatomy, target and fractionation held constant. The mean dose to the arterial mask was 8.67 Gy with intensity-modulated photons and 0.97 Gy with protons, and the mean dose to the venous mask 8.41 Gy and 1.04 Gy respectively, with vessel volumes reproducible to within 4% between the two plans (Figure 3c) [8]. The target coverage was equivalent in both plans, and the reduction is confined to the low-to-intermediate dose region that surrounds the target in the photon plan and is absent distal to the Bragg peak in the proton plan, which is where the large-calibre vessels of the upper abdomen run. This single paired comparison is not evidence of a population effect, but it does show that the differential observed between the subgroups survives when cohort composition is eliminated as an explanation, and it localises the effect to the low-to-intermediate dose region that intensity-modulated photon delivery necessarily deposits outside the target.

Lymphocyte counts followed the dosimetric ordering. Patients treated with photons had lower absolute lymphocyte counts and a greater fall in counts over the course of treatment than patients treated with protons.

### 3.3. Dosimetric Correlates of Lymphopenia

When patients were grouped by whether they developed lymphopenia during treatment rather than by modality, the same two metrics separated them. Mean EDIC was 4.84 Gy in patients who became lymphopenic versus 1.47 Gy in those who did not (*p* = 0.027), and mean IDVe was 3.08 Gy·L versus 0.67 Gy·L, a difference in the same direction that did not reach significance with nine lymphopenic patients (*p* = 0.180) (Figure 4, Table 3). The EDIC value observed in the lymphopenic group falls close to the 4.0 Gy level that separated patients with and without grade 3–4 lymphopenia in VASA Phase I, and the IDVe value is well above the 1 Gy·L level that showed the same trend in that earlier cohort.

### 3.4. Anatomical Gradient Within the Proton Cohort

Restricting the analysis to the 30 patients treated with protons removes the modality effect and exposes the contribution of target location. EDIC and IDVe increased monotonically from brain to head-and-neck to thorax–abdomen targets: EDIC 0.81, 1.11 and 2.17 Gy; IDVe 0.13, 0.26 and 1.63 Gy·L. The integral dose to the target did not follow the same ordering (ID-TV 10.10, 7.80 and 22.57 Gy·L), because head-and-neck targets were the smallest in this cohort (Figure 5, Table 4). The brain–thorax/abdomen difference reached significance for EDIC (*p* = 0.024) and IDVe (*p* = 0.005) but not for ID-TV (*p* = 0.073); for both IDVe and ID-TV the head-and-neck group also differed from the thorax–abdomen group (*p* = 0.007 and *p* = 0.034). The gradient in IDVe is steeper than the gradient in ID-TV: moving from a brain to a thorax–abdomen target roughly doubles the integral dose delivered in the target but increases the energy deposited in the vascular tree more than twelve-fold, which indicates that vascular exposure is governed by the vascular content of the irradiated region and not simply by the size of the target. Because ID-TV is itself the product of mean target dose and target volume, the two quantities cannot be separated from it, and target size is a plausible confounder of the regional gradient. Mean clinical target volume was 242, 138 and 357 cm^3^ for brain, head-and-neck and thorax–abdomen targets respectively (Table 1), and IDVe correlated with target volume within the proton cohort (Spearman ρ = 0.63, *p* < 0.001); the regional ordering of IDVe nevertheless persisted when target volume was entered as a covariate (F = 18.96, *p* < 0.001), and head-and-neck targets, which were the smallest in the cohort, carried a higher IDVe than the larger brain targets. Prescription dose and fractionation also differed between regions (Table 1), and in a cohort of this size their contribution cannot be separated from that of anatomical location.

### 3.5. Lymphocyte Kinetics

Across the whole proton cohort, the absolute lymphocyte count fell from 1.92 to 1.43 × 10^3^/µL between the start and the end of treatment, a mean loss of 0.50 × 10^3^/µL (95% CI 0.29–0.71; *p* < 0.001). The decline was not uniform across target regions. Brain irradiation produced a fall of 0.37 × 10^3^/µL (1.94 to 1.57 × 10^3^/µL; 95% CI 0.08–0.66, *p* = 0.018); head-and-neck irradiation a fall of 0.50 × 10^3^/µL (1.86 to 1.36 × 10^3^/µL; 95% CI 0.15–0.84, *p* = 0.009); and thorax–abdomen irradiation the largest fall, 0.71 × 10^3^/µL (2.02 to 1.31 × 10^3^/µL), which with seven patients did not reach significance (95% CI −0.02 to 1.44, *p* = 0.056). The three regional losses did not differ detectably from one another (all pairwise *p* ≥ 0.32), so the apparent gradient in Δ lymphocytes is not supported by this cohort (Figure 6, Table 5).

The lower of the two recorded lymphocyte counts followed the same ordering but did not separate the regions: 1.53 × 10^3^/µL for brain, 1.32 × 10^3^/µL for head and neck and 1.23 × 10^3^/µL for thorax–abdomen, with no pairwise comparison reaching significance (*p* = 0.319, 0.102 and 0.508). No region reached a mean value below the lower limit of normal. For the proton cohort as a whole the mean of these lowest values was 1.38 × 10^3^/µL, so that the population-level decrease in lymphocytes did not translate into mean lymphopenic values—the depletion is real and statistically significant, but at the doses achieved with protons it remains, on average, within the normal range. Because sampling was tied to the fraction of the course completed rather than to a fixed calendar interval, the two timepoints correspond to different elapsed times in patients receiving short and long courses: the median interval from the first fraction to the final sample was 5 weeks overall, and 5, 6 and 5 weeks for brain, head-and-neck and thorax–abdomen targets. The lower value was recorded at the final timepoint in 24 of 30 patients, so the value reported here is in most patients the count at the end of irradiation rather than a true minimum; with only two samples per patient no nadir can be located at all, and lymphocyte loss during radiotherapy follows patient-specific kinetics that a two-point schedule cannot resolve [71]. Restricting the regional comparison to the 26 patients whose final sample fell between weeks 4 and 6 preserved the ordering of IDVe reported above (0.13, 0.26 and 1.78 Gy·L). A denser sampling schedule extending beyond the end of treatment is required to establish whether the regional differences reflect the depth of depletion, the time at which it is measured, or both.

### 3.6. Lymphocyte Subpopulations

Immunophenotyping showed that the loss of lymphocytes was not restricted to a single lineage. All subpopulation values in this section are percentages of total leucocytes measured by flow cytometry, not absolute cell concentrations; absolute subset counts were not derived, so a fall in a percentage reflects a change in the composition of the leucocyte pool and not necessarily a loss of that lineage in absolute terms. For the proton cohort as a whole (*n* = 30), B cells fell from 3.46% to 2.21% (*p* = 0.009), T cells from 23.03% to 17.15% (*p* = 0.002) and NK cells from 4.89% to 3.57% of leucocytes (*p* = 0.010), and all three decreases were statistically significant (Figure 7, Table 6). Brain irradiation (*n* = 11) left all three subpopulations essentially unchanged (B *p* = 0.514, T *p* = 0.369, NK *p* = 0.429), mirroring the absence of a significant fall in total counts in that group.

The regional pattern of subpopulation loss did not, however, follow the regional pattern of total lymphocyte loss. Although the thorax–abdomen group (*n* = 7) showed the largest mean fall in absolute counts, the significant reductions in T cells (23.02% to 15.03%) and NK cells (5.28% to 3.02%) occurred in the head-and-neck group (*n* = 12). The thorax–abdomen group showed the largest proportional fall in B cells (4.23% to 1.60%), but this did not reach significance in a group of seven patients. Head-and-neck irradiation therefore appears to affect the composition of the circulating lymphocyte pool more than thoracic and abdominal irradiation does, even though the latter depletes it more in absolute terms. With 7 to 12 patients per region and percentages rather than absolute counts as the measured quantity, these subgroup results carry wide uncertainty—the 95% confidence intervals for each start-to-end change are given in Table 6—and the apparent dissociation between the magnitude and the composition of the loss is reported as an observation to be tested, not as an established regional effect.

## 4. Discussion

This study analyses whether the vascular compartment—the anatomical structure that actually transports the irradiated lymphocyte pool—can be imaged, delineated and dosimetrically characterised within a routine clinical workflow, and whether the resulting metrics carry information about radiation-induced lymphopenia. Three findings emerge, and they sit at different levels of evidence. The first is a demonstration; the second and third are associations observed in an unmatched, single-institution cohort without mature clinical endpoints, and are therefore hypotheses. First, patient-specific vascular segmentation from flow-contrast MRI is feasible at simulation and yields a vessel DVH for every plan at the cost of a single additional acquisition. Second, the resulting integral dose to vessels separates irradiation modalities more sharply than EDIC does, and separates lymphopenic from non-lymphopenic patients. Third, within a single modality, the anatomical location of the target drives both vascular dose and the magnitude of lymphocyte depletion, but the lineage composition of that depletion follows a different regional pattern from its magnitude.

### 4.1. Making the Vascular Compartment Visible

The dominant approach to blood dosimetry has been computational: simulate blood transport through a time-resolved dose distribution using reference vascular anatomy and physiological flow models [29,30,31,32,33]. These frameworks are sophisticated and have produced results that translate directly into planning decisions, including the demonstration that prolonging fraction delivery time increases the dose to circulating lymphocytes [34] and the correlation of dynamic blood dose with clinical outcome in head-and-neck cancer [35]. On the other hand, the FLIP approach has included patient-specific information [38] and FLIP-HEDOS has extended the original generic framework to consider blood recirculation and patient-specific compartments [39]. Their principal uncertainty is the anatomy they assume, and the magnitude of that uncertainty has been quantified: inter-subject cerebrovascular variability alone materially changes the computed dose to circulating blood cells in glioblastoma [37]. The approach reported here is complementary rather than competing. It does not simulate transport; it measures where the vessels are in this patient and how much dose they receive, producing a quantity—IDVe—that is defined on the individual anatomy and computed by the treatment-planning system with no additional modelling assumptions. In principle the two approaches converge: patient-specific vascular masks of the kind generated here are precisely the geometric input that blood-transport models currently have to approximate.

The feasibility result matters on its own terms. Reviews of lymphocyte-sparing radiotherapy consistently identify the absence of a delineable structure as the practical obstacle to writing dose constraints for the circulating compartment [56,62,72]. The surrogates in current use—spleen [57,58], thymus [59,60], bone marrow [61]—are lymphocyte reservoirs, and constraining them addresses only part of the problem, because a lymphocyte that is irradiated while in transit through a femoral vein is not protected by sparing the spleen. A segmented vascular tree is the missing structure, and it behaves in the planning system like any other organ at risk: it can be contoured, it produces a DVH, and it can therefore in principle be entered into optimisation as an objective.

The two metrics reported here are not interchangeable, and the question of what the integral dose to vessels adds to EDIC deserves an explicit answer. EDIC is a closed-form estimate of the equivalent uniform dose absorbed by the whole blood pool, reconstructed from the mean doses to the lung, heart and liver, the integral body dose and the number of fractions [23,24]; it is therefore a model of the circulating compartment in which the patient’s own vascular anatomy enters only through the dose to three reference organs, and its coefficients were derived principally in thoracic cohorts. IDVe is a measurement rather than a model: it is the product of the mean dose and the volume of a structure that has been delineated on the individual patient, computed by the treatment-planning system with no further assumptions, and it is available for any anatomical region without recalibration. The two quantities answer different questions—EDIC estimates what the blood pool absorbs on average, IDVe quantifies the energy deposited in the conduits that carry it—and they are expected to be correlated, because both scale with the irradiated volume and the delivered dose. In this series they were correlated but not interchangeable: IDVe separated the irradiation modalities and the lymphopenic from the non-lymphopenic patients more widely than EDIC did, and its regional gradient within the proton cohort was steeper. The most plausible reading is that they are complementary, with a shared component reflecting irradiated volume and an independent component in which IDVe carries the patient-specific vascular geometry that EDIC averages away; whether that independent component also carries independent predictive information for lymphopenia cannot be settled in a cohort of this size, and quantifying the correlation between the two metrics and their joint contribution to a prediction model is a defined objective of the next phase. Neither metric encodes blood flow or the spatial distribution of dose within the vascular tree, which is the gap that patient-specific transport models are designed to fill [38,39].

### 4.2. Modality Differentials in Context

The approximately four-and-a-half-fold reduction in EDIC and seven-fold reduction in IDVe with protons is consistent in direction and broadly in magnitude with the published comparative literature. Lower rates of severe lymphopenia with proton beam therapy than with IMRT have been reported in propensity-matched oesophageal cohorts [40,41], in comparative risk modelling [42], in the comprehensive analysis of a randomised phase IIB trial [43] and in multi-institutional medulloblastoma series [44]; EDIC specifically has been shown to fall with protons in mediastinal Hodgkin lymphoma [45] and breast cancer [44], and technique-dependent immune sparing has been demonstrated in postoperative settings [49]. Carbon ions extend the same logic further in pancreatic cancer [47]. What the present data add is that the differential is visible not only in the modelled blood-pool dose but in the measured energy deposited in the patient’s own vascular tree, and that the vascular metric discriminates more strongly than the blood-pool metric does. That is a mechanistically coherent result: the absence of exit dose removes precisely those low-dose volumes through which large-calibre vessels commonly run. The most direct evidence comes from the randomised phase II comparison of protons with photons in glioblastoma, in which proton therapy reduced the likelihood of high-grade radiation-induced lymphopenia [73], and from comparative series in stage III lung cancer in which scanning-beam proton therapy reduced both dosimetric and haematological burden relative to photon IMRT [74]. Within the VASA project itself, an interim analysis of the first 30 patients of this cohort gave a mean EDIC of 5.18 Gy with photons versus 1.33 Gy with protons and a mean IDVe of 4.92 versus 0.40 Gy·L [8]; the present values in 39 patients reproduce that ordering and magnitude as recruitment has continued.

This comparison must be read with its design in mind. The photon subgroup comprises nine patients from an open recruitment stream, is not matched to the proton cohort, and differs from it in the distribution of tumour sites and in the clinical and treatment variables compared in Table 1. The observed differential is therefore an indication of the direction and approximate scale of the effect, not an effect estimate. The direction of the likely bias can be reasoned about even if its size cannot be estimated. Because thoracic and upper-abdominal targets are over-represented among the photon-treated patients, part of the measured modality differential is attributable to anatomy rather than to beam quality—the same anatomical gradient that Section 3.4 demonstrates within the proton cohort, where modality is held constant, would by itself raise EDIC and IDVe in the photon group. Prescription dose, fractionation and the use of concurrent systemic therapy differ between the groups in the same direction, and each of them independently influences lymphocyte counts, so the subgroup difference in lymphocyte endpoints cannot be attributed to vascular dose alone. The paired replanning of a single patient (Figure 3c) is the one comparison in this report in which anatomy, target and fractionation are held constant, and the differential it shows is of the same order as the subgroup differential, which is reassuring but is a single case. Until the photon stream is closed and a site- and dose-matched comparison is possible, the magnitude of the modality effect reported here should be treated as an upper bound rather than as an estimate. The randomised comparison of intensity-modulated proton therapy with IMRT in oropharyngeal cancer [51] illustrates the level of evidence required before modality differentials in immune endpoints can be treated as established, and the counterfactual modelling approaches now being developed to individualise modality selection [75] indicate how such decisions may eventually be made prospectively.

### 4.3. Anatomy Determines Vascular Exposure

Within the proton cohort, where modality is held constant, target location drove every dosimetric endpoint. The nearly nine-fold increase in IDVe between brain and thorax–abdomen targets, against a roughly two-fold increase in ID-TV, shows that vascular exposure is not a proxy for target size. The thorax and upper abdomen contain the heart, the great vessels, the pulmonary circulation and the portal system; a target in that region unavoidably places a large, high-flow vascular volume in or near the irradiated field, which is the same anatomical reasoning that underlies the terms of the EDIC formulation itself [23,24] and the observation that thoracic and upper-abdominal tumours account for much of the reported burden of severe lymphopenia [9,12]; the size of the irradiated field has itself been shown to govern the risk and severity of treatment-induced lymphopenia [76]. Conversely, the brain is a low-flow, low-volume vascular target relative to the thorax, and cerebral irradiation with protons produced neither a significant fall in lymphocyte counts nor a significant change in any subpopulation in this series—despite the fact that glioma cohorts treated with photons show some of the most severe lymphopenia in the literature [16,17,22]. The contrast supports the interpretation that it is the irradiated vascular volume, rather than the irradiated tissue type, that determines the immune cost of a plan. These results are compatibles with the results obtained in silico models FLIP [38] and FLIP-HEDOS [39], where the irradiated volume and their specific location related to the tumour are relevant parameters.

### 4.4. Magnitude and Composition of Lymphocyte Loss Diverge

The most unexpected observation in this series is the dissociation between where lymphocytes are lost in greatest number and where the composition of the circulating pool changes most. Thorax–abdomen targets produced the largest mean fall in absolute counts, yet that fall did not reach significance in seven patients, while the significant reductions in T and NK subpopulations occurred in head-and-neck targets. Several explanations are plausible and are not mutually exclusive. None of them is tested by the present data, and they are offered as candidate mechanisms to be examined prospectively rather than as demonstrated causes; the observation itself rests on subgroups of 7 to 12 patients and on percentages rather than absolute subset counts. Head-and-neck irradiation, particularly the bilateral neck volumes used in a third of that group, exposes the cervical lymph node chains and the carotid–jugular axis, so that both resident and transiting lymphocytes are irradiated, and irradiation of lymphoid volumes has been specifically associated with lymphopenia in head-and-neck cancer [62]. Subpopulations also differ in intrinsic radiosensitivity and in repopulation kinetics, so the same absolute loss can leave different compositional signatures depending on which compartment is drained [3,4,5,77]. Finally, part of the effect may be temporal: the two-timepoint sampling schedule used here captures depletion during treatment but not the subsequent recovery, and lineages recover at different rates [78,79]. Distinguishing these mechanisms requires longer follow-up and CD4/CD8 resolution, both of which are planned for the next phase of the project. The third explanation has direct experimental support: blood cell populations differ in DNA repair capacity and in intrinsic radiosensitivity, so an identical absorbed dose does not deplete B, T and NK compartments in equal proportion [5,80]. The host genotype adds a further layer to this, and one that the present design cannot control for: in a genetically diverse murine population the acute and persistent depletion of individual immune compartments after irradiation segregates as a heritable trait with compartment-specific genetic architecture [6], and in patients the intrinsic radiosensitivity of peripheral lymphocytes varies between individuals and correlates with the severity of late normal-tissue reactions [7]. Inter-individual genetic variation is therefore expected to contribute to the dispersion of lymphocyte endpoints within each of our anatomical groups, and in a cohort of this size it cannot be distinguished from a regional effect; germline characterisation alongside serial immunophenotyping would be required to separate the two. A regional difference in which vascular territory is irradiated might therefore be expected to produce a regional difference in the composition, and not merely the size, of the surviving lymphocyte pool. That expectation is consistent with what we observed, but consistency is not demonstration: establishing that anatomical location independently determines the pattern of immune-cell depletion requires absolute subset counts, larger and balanced regional groups, follow-up through the recovery phase and adjustment for the systemic-therapy and genetic factors discussed above.

### 4.5. Implications for Treatment Planning

Making vascular dose measurable is a precondition for acting on it, but it is not by itself a reason to act. Four possible consequences follow from this work, set out in increasing order of ambition and of the evidence each would require before it could be adopted. Only the first is supported by the present data:

- Measurement: Vessel masks can be generated at simulation and reported alongside conventional OAR DVHs, so that the immune cost of a plan becomes a visible quantity rather than an inference.
- Paradigm: Whether circulating blood and vascular structures warrant consideration as structures of interest in their own right, on the same footing as the parallel organs whose dose is routinely constrained, is a question this work raises rather than answers. It would be premature to place them on that footing before it is known what the measured dose predicts.
- Candidate thresholds: Values that might eventually serve as thresholds can be read off the present and earlier data—EDIC in the region of 4 Gy, from VASA Phase I and consistent with published EDIC–outcome relationships [24,25,26], and IDVe in the region of 1 Gy·L—but neither is a dose constraint, and neither should be used as one. Both were derived from the cohorts that generated them, with no independent validation and no dose–response modelling; formal constraint derivation requires the larger cohorts that systematic reviews of the field have called for [56] together with normal-tissue complication probability modelling of the kind now being applied to blood dose [81].
- Optimisation: If a vessel structure were ever to carry a validated constraint, plan optimisation could in principle trade vascular dose against other objectives. In silico work in pelvic [82] and head-and-neck [83] radiotherapy suggests that blood-dose reductions are achievable without compromising target coverage, and complementary strategies—shorter beam-on times [34], beam arrangements that avoid high-flow structures, and ultimately ultra-high dose rate delivery [84]—act on the same variable. This remains the most speculative of the four, and the present study provides no evidence for it.

Two obstacles stand between a measurable vascular dose and a usable planning objective, and neither is addressed by the data reported here. The first is the cost of the trade. Clinical plans already balance a long list of competing dose–volume objectives, and in the anatomical regions where vascular dose is highest the vascular tree is interleaved with the very organs whose constraints are most binding—the heart and great vessels in the thorax, the carotid and jugular axis alongside the spinal cord, parotids and constrictors in the neck, the portal and mesenteric circulation alongside bowel, liver and kidney in the abdomen. Adding a vascular objective to such a plan is not a free operation: dose displaced away from vessels has to go somewhere, and whether an achievable reduction in IDVe can be obtained without degrading target coverage or relaxing an established organ-at-risk constraint is an open question that only systematic replanning studies can answer. The in silico results cited above were obtained with blood-dose surrogates rather than with segmented vessel masks, and they do not establish that the same is true of IDVe. The second obstacle is priority. Even if vascular dose can be reduced at an acceptable cost, it is not known where a vascular objective should rank among existing constraints, because the clinical consequence of a given IDVe has not been quantified: this series relates vascular dose to lymphocyte counts, not to survival, toxicity or infection, and lymphopenia is an intermediate endpoint whose causal position is supported by mediation analysis in one disease site [19] but not established generally. Prioritising a vascular constraint over an established one on the current evidence would not be justified. The appropriate next step is not to optimise against vascular dose but to measure it prospectively alongside clinical endpoints, so that its predictive weight—relative to the constraints already in use—can be established before any planning trade-off is proposed.

The clinical rationale for pursuing this is the interaction between lymphopenia and immunotherapy. Radiotherapy and checkpoint blockade are now routinely combined [20], and the lymphocyte pool is the substrate on which the second of these depends; preclinical and clinical work continues to refine how radiation-induced immunosuppression, including myeloid-derived suppressor cell expansion, shapes that interaction [21,85]. Selecting a plan that preserves immunocompetence is therefore not a secondary consideration but part of the treatment strategy, and prediction models that identify patients at risk of severe lymphopenia before treatment starts [86,87] become more useful when a modifiable dosimetric variable is available to act on.

### 4.6. Limitations

Several limitations constrain the interpretation of these results. The cohort is small and single-institution, and the photon subgroup in particular (*n* = 9) is neither matched nor closed to recruitment, so all modality comparisons are descriptive. Comparisons were made with Welch’s *t*-test for continuous variables and Fisher’s exact test for categorical variables, without correction for multiple comparisons, so the familywise error rate across the comparisons reported here is inflated, and no multivariable model was fitted because the number of patients does not support one; the analyses are consequently hypothesis-generating. Measures of dispersion and confidence intervals are now reported throughout, but with 7 to 12 patients in most subgroups those intervals are wide, and several of the regional differences described above are compatible with no difference at all. Clinical endpoints—progression-free and overall survival, and toxicity—are not yet mature in this cohort, so the study demonstrates that vascular dose tracks lymphocyte depletion but cannot yet show that it tracks outcome. Concurrent systemic therapy was recorded but not modelled, and corticosteroid exposure during irradiation was not captured by the study database at all; both are established determinants of lymphocyte counts. Stage, treatment intent, prior surgery, the specific delivery technique and gross tumour volume were likewise not recorded, so Table 1 characterises the two modality groups on the variables that were collected rather than on the full set a matched comparison would require. On the technical side, all vessel masks were produced by a single observer and no second delineation was performed, so the reproducibility of the segmentation—and hence of IDVe—is unquantified; given the interobserver variability documented for conventional organs at risk [67], this is a material gap that a multi-observer study must close. The MSUM-based segmentation is further limited by partial-volume effects at vessel calibres approaching the 1–1.7 mm voxel size [68], so the mask represents the large-vessel compartment rather than the whole vascular volume and the smallest vessels—which collectively carry a substantial fraction of the blood volume and the entire exchange surface—are not represented; and the EDIC formulation applied here was derived principally in thoracic cohorts, which introduces an approximation when it is applied to brain and head-and-neck targets. Finally, IDVe as defined is an integral quantity and does not encode the spatial distribution of dose within the vascular tree or the flow rate through the irradiated segments, both of which determine how much of the circulating pool is actually exposed. The cohort also departs from the original protocol design, which specified ten patients for each of six anatomical sites; recruitment in practice followed clinical referral patterns, so brain, head-and-neck and thorax–abdomen targets dominate and pelvis, extremity and breast targets are not represented. The sampling schedule is a further limitation: two timepoints keyed to the fraction of the course completed cannot locate a nadir at all, and because course length varies between anatomical regions the regional comparison of the lowest recorded values confounds the depth of depletion with the time at which it was measured. Subpopulations were measured as percentages of leucocytes rather than as absolute concentrations, which limits what can be inferred about the loss of any individual lineage. Finally, the paired replanning comparison in Figure 3c is a single case included to illustrate the dosimetric mechanism, and no paired series exists yet.

### 4.7. Future Directions

The immediate priorities are to close recruitment of the photon cohort so that a matched comparison becomes possible, to extend sampling beyond the end of treatment in order to capture recovery kinetics, to add a second independent delineation so that the reproducibility of the vessel mask can be quantified, and to resolve T cells into CD4 and CD8 compartments. Beyond that, the natural development is to couple the patient-specific vascular masks generated here to the blood-transport models that currently rely on reference anatomy [29,30,36], producing a dose to circulating lymphocytes that is both dynamic and anatomically individualised. Validation of candidate IDVe thresholds in a larger prospective series, and only then their prospective use as optimisation objectives, would complete the translation from measurement to intervention. The present cohort also indicates where such a series should be conducted. A uniform-cohort study would gain most by selecting a disease site in which the irradiated vascular burden is high, the prescription is standardised, lymphopenia is frequent enough to be an informative endpoint, and both modalities are realistically available. Locally advanced oesophageal cancer meets all four conditions most completely: the target encircles the descending aorta and azygos system, chemoradiation schedules are highly standardised, severe lymphopenia is common, and the proton-versus-photon comparison has already been taken to a randomised endpoint [43]. Stage III non-small-cell lung cancer is the natural second choice, given the thoracic vascular burden and the existing EDIC–survival relationship established in NRG/RTOG 0617 [24]. Locally advanced pancreatic cancer offers the highest vascular density of any abdominal target, with the coeliac axis and superior mesenteric vessels inside or adjacent to the target volume [47]. At the opposite end of the range, oropharyngeal cancer treated with bilateral neck volumes is the site in which the compositional changes observed here were most pronounced and in which a randomised modality comparison already exists [51], which makes it the appropriate setting for the subset-resolved question rather than the magnitude question. Glioma, by contrast, is informative chiefly as a low-vascular-burden control. We would propose oesophageal or stage III lung cancer for the primary validation of IDVe thresholds, with oropharyngeal cancer as the setting for the immunophenotypic question. Two further directions follow from the biology rather than the dosimetry. Serum markers of endothelial injury and the inflammatory and immune biomarkers catalogued for radiation response [55,88] would allow the vascular wall and the circulating pool to be monitored as distinct compartments, which matters because the endothelial dose–response governs long-term vascular morbidity while the circulating dose governs immunocompetence. And if vascular sparing does preserve immunocompetence, the effect should be detectable in the systemic antitumour responses that radiotherapy can elicit [89]; delivery regimens that compress beam-on time, including FLASH dose rates, are being examined for exactly this reason [90]. New methodologies (FLIP, FLow and Irradiation Personalized) involve the use of patient-specific vasculature, a blood flow velocity field, and dose delivery dynamics recovered from the irradiation equipment. The combination of these tools with biological data will provide the community with a valuable tool to predict and potentially avoid damage to blood cells.

## 5. Conclusions

Blood vessels can be segmented from flow-contrast MRI acquired at simulation, imported into the treatment-planning system as an organ at risk and characterised dosimetrically in routine clinical practice. That feasibility, demonstrated in all 39 patients of this prospective series, is the primary result of the study. In the same series the resulting integral dose to vessels, together with the effective dose to circulating immune cells, was approximately seven- and four-and-a-half-fold lower with protons than with photons, was higher in patients who developed lymphopenia than in those who did not, and followed a monotonic anatomical gradient from brain through head-and-neck to thorax–abdomen targets that persisted after adjustment for target volume; the corresponding gradient in the magnitude of lymphocyte depletion was in the same direction but did not reach significance between regions. These associations were obtained in unmatched subgroups of 7 to 12 patients without correction for multiple comparisons and without clinical endpoints, and are therefore exploratory.

A systematic study of the impact of radiation on vessels and circulating blood in clinical practice could therefore open up new opportunities for the optimisation of radiotherapy treatment planning, and the measurements reported here provide the quantities on which such a study would be built. Whether minimising the irradiation of vascular structures at low and intermediate doses actually preserves immunocompetence, at what cost to competing organ-at-risk objectives, and with what clinical benefit, are open questions that this work is designed to make testable rather than to answer. Prospective validation in larger, uniform cohorts with mature clinical endpoints is required before vascular dose can be treated as a planning objective.

## Figures and Tables

**Figure 1:**
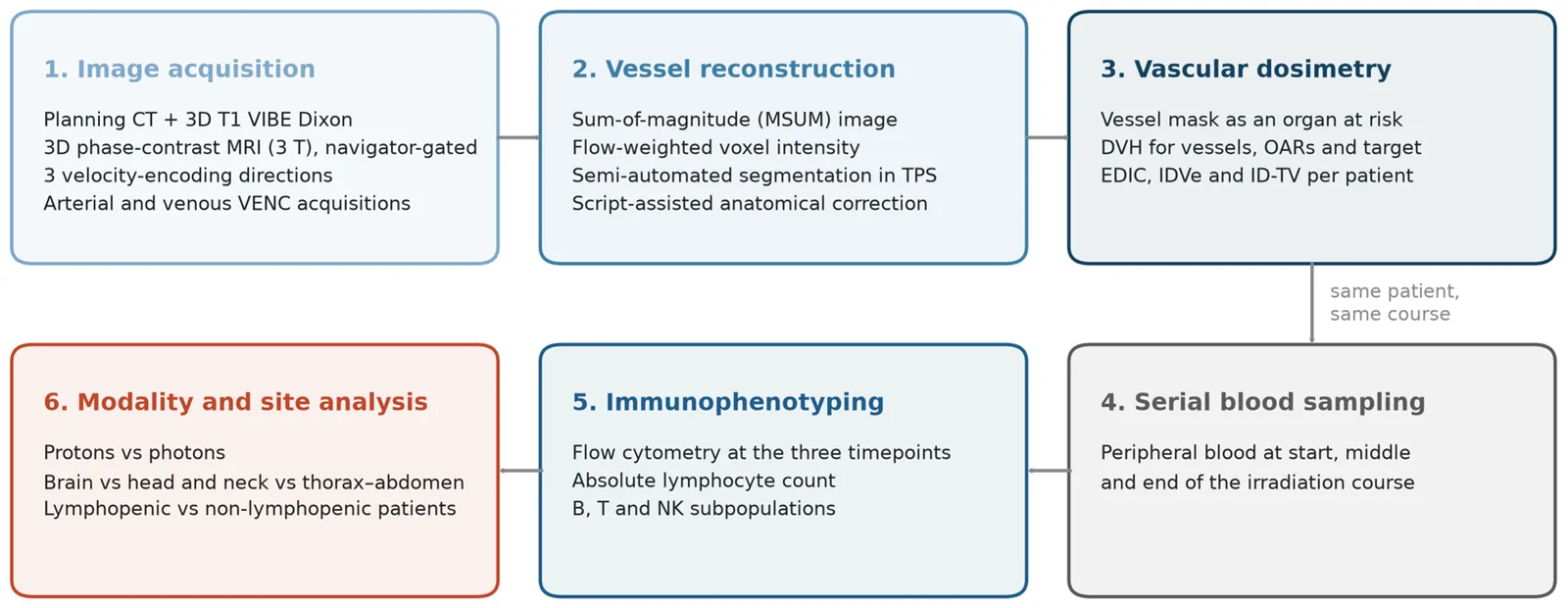
Workflow of VASA Phase II. Flow-contrast MRI acquired at simulation is reconstructed into a sum-of-magnitude (MSUM) image in which voxel intensity scales with flow velocity; the vascular tree is segmented from that image, imported into the treatment-planning system as an organ at risk, and used to generate vessel dose–volume histograms from which EDIC, IDVe and ID-TV are derived. Peripheral blood is sampled at the start, middle and end of the same irradiation course and analysed by flow cytometry, allowing dosimetric and immunological endpoints to be paired within each patient. TPS, treatment-planning system; OAR, organ at risk; DVH, dose–volume histogram; VENC, velocity encoding; EDIC, effective dose to circulating immune cells; IDVe, integral dose to vessels; ID-TV, integral dose to target volume.

**Figure 2:**
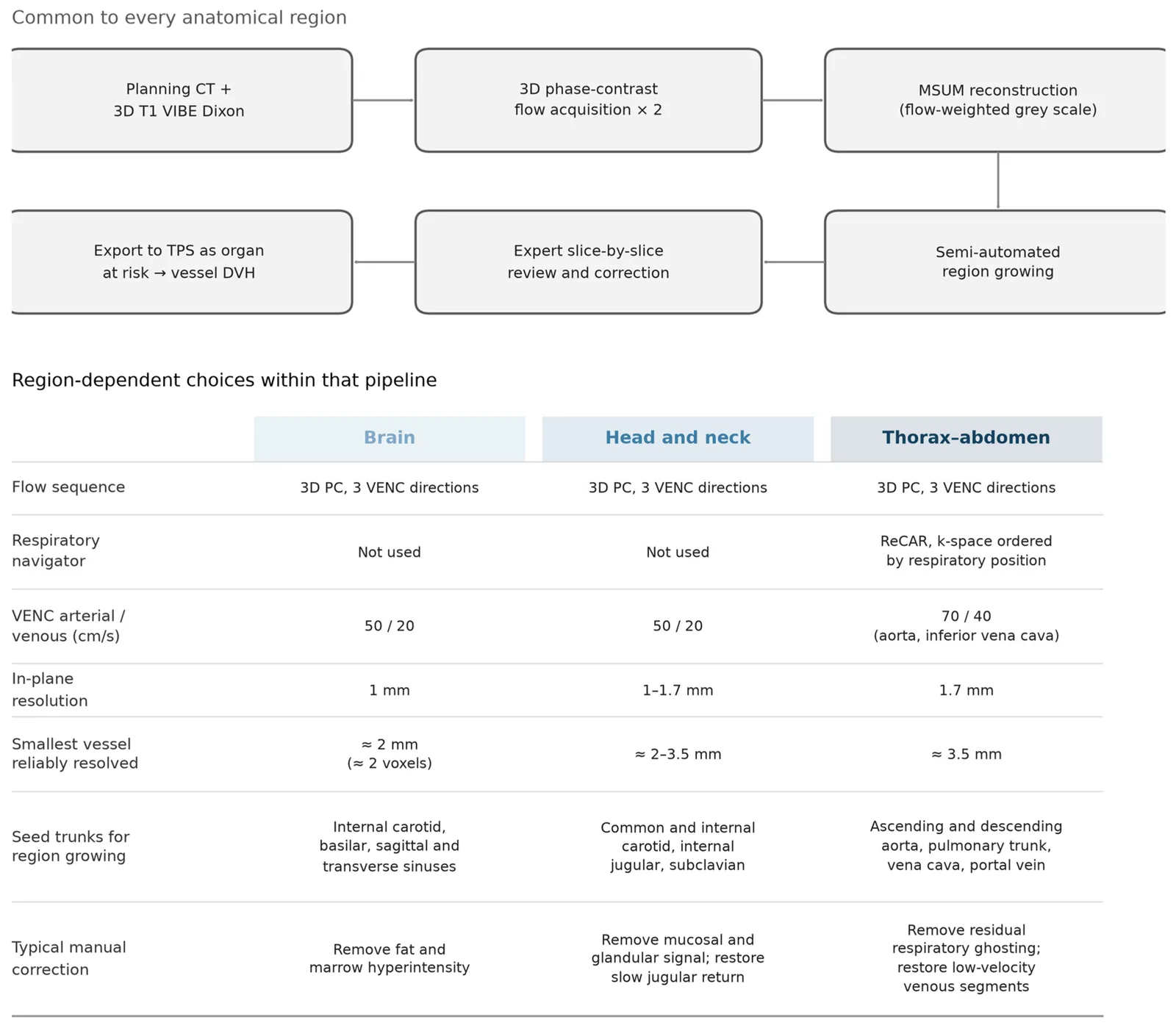
Definition of the vascular organ at risk, and how it differs between anatomical regions. Top: the segmentation pipeline applied to every patient, from the simulation acquisitions through the flow-weighted MSUM reconstruction, semi-automated region growing and expert correction, to export into the treatment-planning system as an organ at risk from which a vessel dose–volume histogram is generated. Bottom: the parameters of that pipeline that were chosen per anatomical region, including the velocity-encoding values, the use of the respiratory navigator, the spatial resolution and hence the smallest vessel calibre reliably resolved, the vascular trunks used to seed region growing, and the manual corrections typically required. The calibre limit is the practical consequence of partial-volume averaging at the acquired voxel size and defines the large-vessel compartment that IDVe quantifies. PC, phase contrast; VENC, velocity encoding; MSUM, magnitude sum; TPS, treatment-planning system; DVH, dose–volume histogram; ReCAR, respiratory-position-driven navigator.

**Figure 3:**
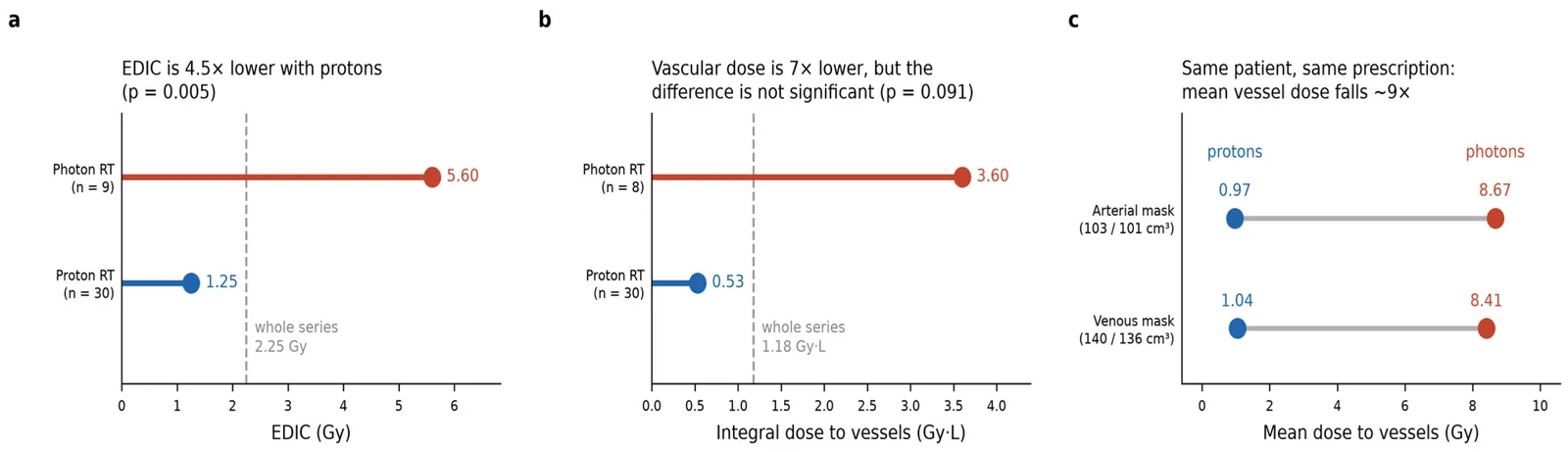
Dose to the circulating immune compartment by irradiation modality. (**a**) Effective dose to circulating immune cells (EDIC) and (**b**) integral dose to the MRI-segmented vascular tree (IDVe), as group means for the photon (*n* = 9) and proton (*n* = 30) subgroups; the dashed line marks the whole-series mean (*n* = 39). (**c**) Paired in silico comparison in a single representative patient with an upper-abdominal target, replanned for both modalities at the same prescription (40 Gy in 20 fractions) on the same anatomy and the same vessel masks; values are the mean dose to each vessel structure, with the structure volumes given in parentheses for the photon and proton plan respectively. Panels (**a**,**b**) compare unmatched subgroups and should be read as the direction and approximate scale of the differential rather than as an effect estimate; panel (**c**) removes the cohort-composition confound but is a single case and carries no statistical weight.

**Figure 4:**
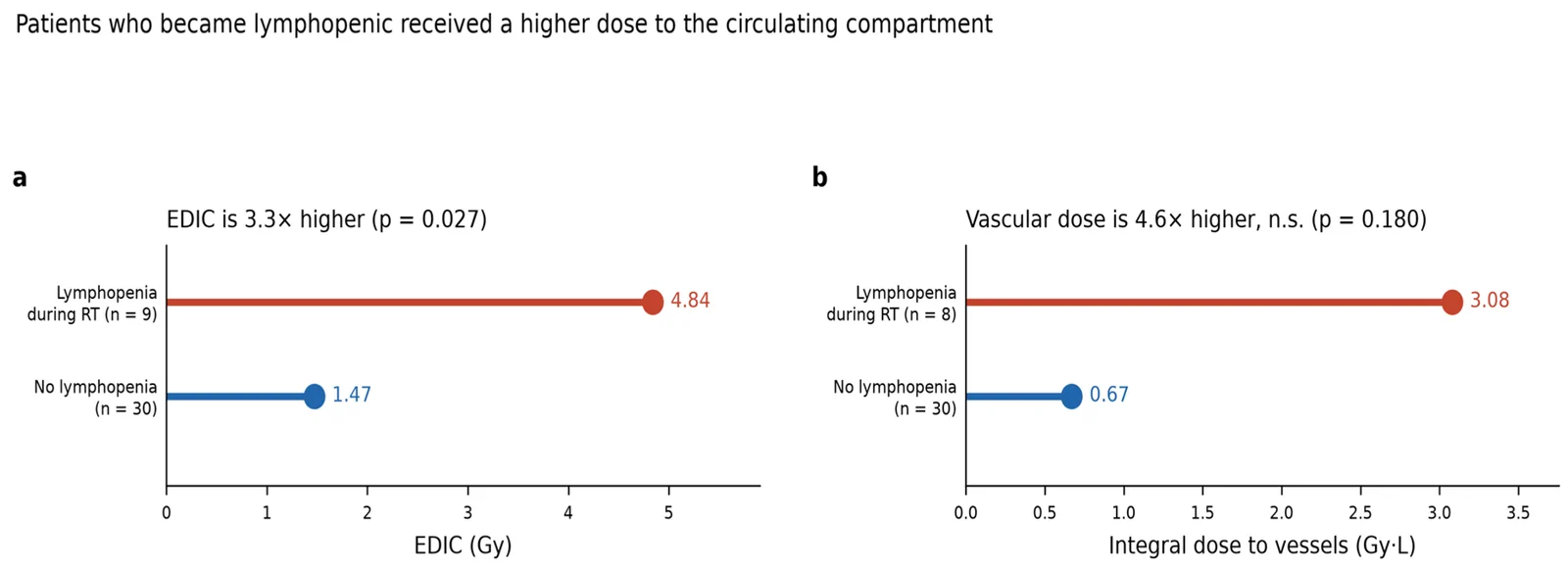
Vascular and circulating-blood dose in patients who did and did not develop lymphopenia during radiotherapy. (**a**) Effective dose to circulating immune cells; (**b**) integral dose to vessels. Points are group means across the whole series (*n* = 39); lymphopenia is defined as a recorded ALC below 0.8 × 10^3^/µL (CTCAE grade ≥ 2), reached by 9 of 39 patients.

**Figure 5:**
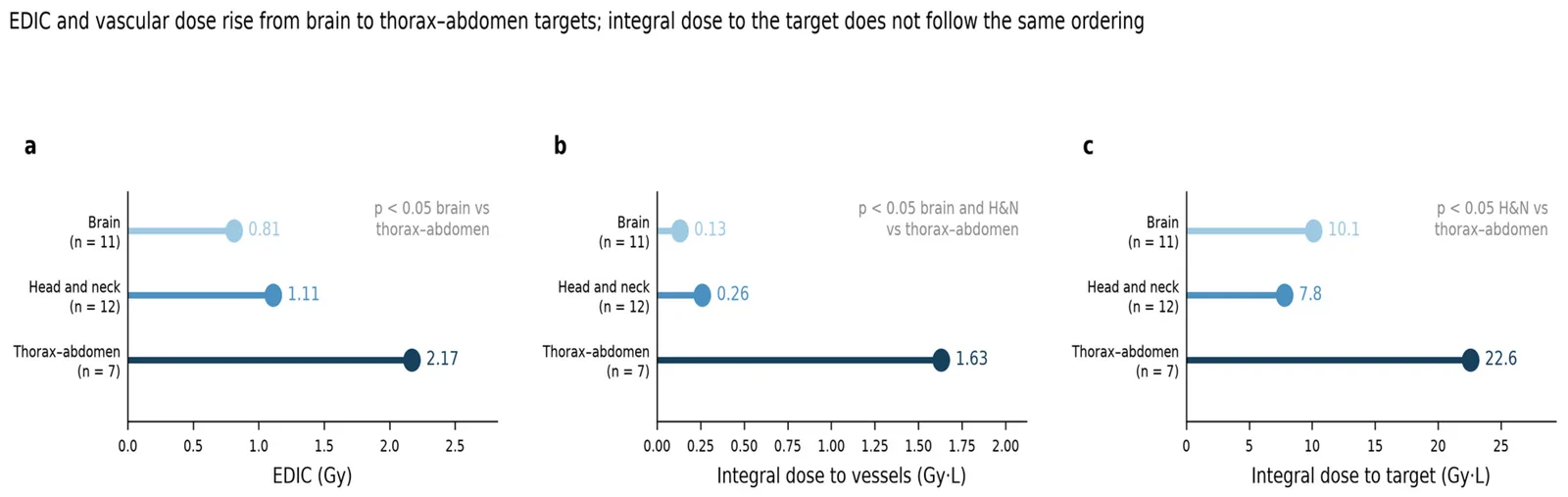
Dosimetric metrics by anatomical target region in the proton cohort (*n* = 30). (**a**) Effective dose to circulating immune cells; (**b**) integral dose to vessels; (**c**) integral dose to target volume. Points are group means; pairwise comparisons by Welch’s *t*-test. Note the independent *x*-axis scale in each panel. H&N, head and neck.

**Figure 6:**
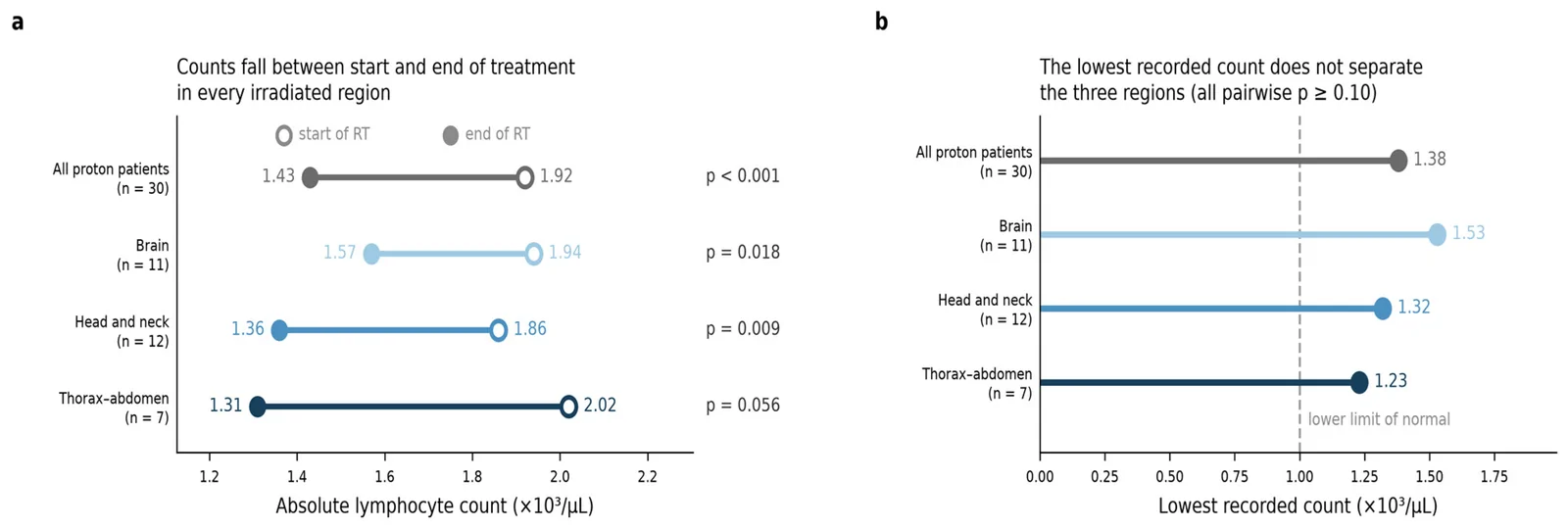
Lymphocyte kinetics by anatomical target region in the proton cohort. (**a**) Absolute lymphocyte count at the start and at the end of the irradiation course; (**b**) the lower of the two recorded lymphocyte counts. Points are group means; *p*-values in (**a**) from the paired *t*-test comparing start with end within each group. In (**b**), no pairwise comparison between anatomical regions reached significance (Welch’s *t*-test, all *p* ≥ 0.10).

**Figure 7:**
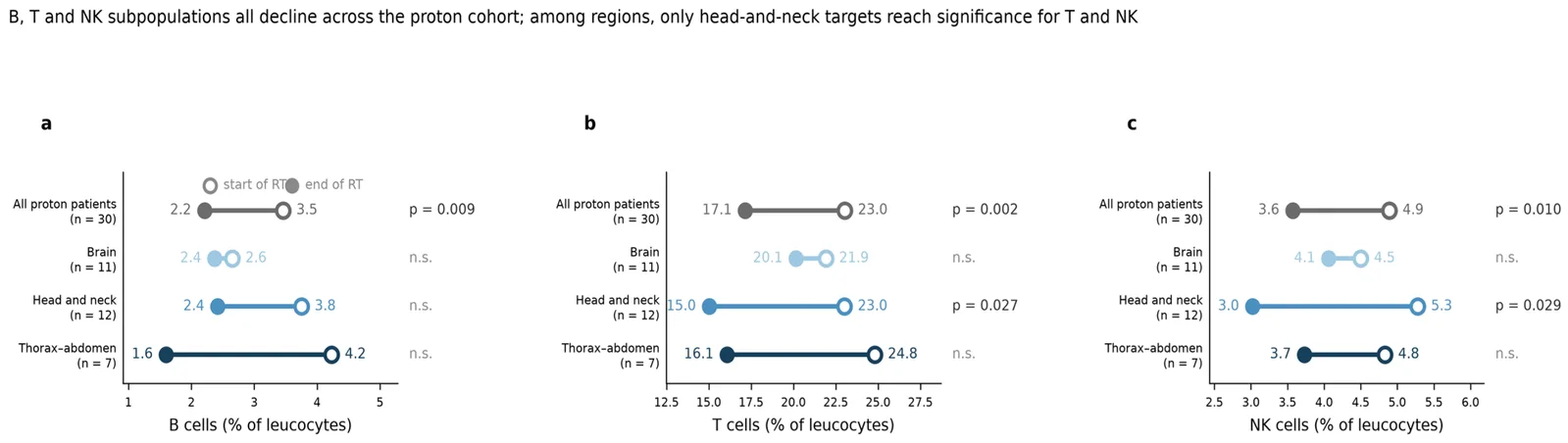
Lymphocyte subpopulations at the start and at the end of the irradiation course, by anatomical target region in the proton cohort. (**a**) B cells; (**b**) T cells; (**c**) NK cells, each as a percentage of leucocytes. Open markers, start of radiotherapy; filled markers, end of radiotherapy. *p*-values from the paired *t*-test comparing start with end within each group. Note the independent *x*-axis scale in each panel. n.s., not significant. The large relative fall in B cells in the thorax–abdomen group (4.23% to 1.60%) does not reach significance at *n* = 7 and should not be read as a negative result.

**Table 1:** Cohort, treatment, dosimetric and immunological characteristics, by irradiation modality. Values are mean ± SD, median (range) or *n* (%). Continuous variables were compared with Welch’s *t*-test; categorical variables with Fisher’s exact test, extended to the Fisher–Freeman–Halton exact test for variables with more than two categories. The minimum expected cell count was 3.0, so the chi-square approximation is not valid in this cohort. EDIC and IDVe are given with both the normal-theory interval and the median, because both distributions are right-skewed. Stage, treatment intent, prior surgery, the specific delivery technique, gross tumour volume, overall treatment time and corticosteroid exposure were not captured in the study database and are therefore not reported; this is stated as a limitation in Section 4.6.

Characteristic	Category	Proton RT (*n* = 30)	Photon RT (*n* = 9)	Whole Series (*n* = 39)	*p* ^1^
Recruitment period	March 2021–May 2022	—	—	—	—
Age (years)	Mean ± SD	46.7 ± 18.0	66.3 ± 18.1	51.2 ± 19.7	0.013
Sex	Female	11 (37%)	1 (11%)	12 (31%)	0.228
	Male	19 (63%)	8 (89%)	27 (69%)	
Target region	Brain	11 (37%)	1 (11%)	12 (31%)	0.014
	Head and neck	12 (40%)	1 (11%)	13 (33%)	
	Thorax–abdomen	7 (23%)	7 (78%)	14 (36%)	
Tumour histology	Glioma/ependymoma	7 (23%)	0 (0%)	7 (18%)	0.017
	Chordoma/sarcoma	11 (37%)	1 (11%)	12 (31%)	
	Squamous/salivary-type carcinoma	6 (20%)	1 (11%)	7 (18%)	
	Adenocarcinoma/other carcinoma	6 (20%)	7 (78%)	13 (33%)	
Vascular comorbidity	Yes	3 (10%)	6 (67%)	9 (23%)	0.002
Toxic habits	Yes	8 (27%)	2 (22%)	10 (26%)	1.000
Prior chemotherapy or radiotherapy	Yes	20 (67%)	6 (67%)	26 (67%)	1.000
Concurrent systemic therapy	Yes	7 (23%)	3 (33%)	10 (26%)	0.669
Prescription dose (Gy)	Median (range)	60 (24–76)	50 (18–69)	60 (18–76)	0.161
Dose per fraction (Gy)	Median (range)	2.2 (1.3–8.0)	2.0 (1.2–3.0)	2.2 (1.2–8.0)	
Number of fractions	Median (range)	30 (5–34)	25 (10–30)	25 (5–34)	0.248
Clinical target volume (cm^3^)	Mean ± SD	227.3 ± 281.3	460.2 ± 429.5	281.0 ± 330.3	0.156
	Brain	242.4 ± 387.9	1446.0 (*n* = 1)	342.7 ± 507.5	
	Head and neck	137.5 ± 167.9	73.0 (*n* = 1)	132.5 ± 161.7	
	Thorax–abdomen	357.4 ± 209.2	374.7 ± 224.6	366.1 ± 208.7	
EDIC (Gy)	Mean ± SD	1.25 ± 1.10	5.60 ± 3.48	2.25 ± 2.63	0.005
	95% CI	0.84–1.66	2.92–8.27	1.40–3.10	
	Median (range)	0.83 (0.09–4.20)	4.23 (1.54–12.74)	1.47 (0.09–12.74)	
IDVe (Gy·L)	Mean ± SD	0.53 ± 0.79	3.60 ± 4.43	1.18 ± 2.41	0.091
	95% CI	0.23–0.83	−0.11–7.30	0.38–1.97	
	Median (range)	0.18 (0.00–2.96)	2.30 (0.23–13.73)	0.25 (0.00–13.73)	
ID-TV (Gy·L)	Mean ± SD	12.09 ± 11.90	18.59 ± 13.01	13.59 ± 12.30	0.204
Baseline ALC (×10^3^/µL)	Mean ± SD	1.92 ± 0.47	1.30 ± 0.57	1.78 ± 0.55	0.012
	95% CI	1.75–2.10	0.87–1.74	1.60–1.96	
End-of-treatment ALC (×10^3^/µL)	Mean ± SD	1.43 ± 0.48	0.67 ± 0.30	1.25 ± 0.55	<0.001
Lowest recorded ALC (×10^3^/µL)	Mean ± SD	1.38 ± 0.43	0.67 ± 0.30	1.21 ± 0.50	<0.001
Baseline lymphopenia	ALC < 1.0 × 10^3^/µL	0 (0%)	3 (33%)	3 (8%)	0.009
Lymphopenia during RT	CTCAE grade ≥ 2	2 (7%)	7 (78%)	9 (23%)	<0.001
Worst CTCAE grade	Grade 0	25 (83%)	1 (11%)	26 (67%)	<0.001
	Grade 1	3 (10%)	1 (11%)	4 (10%)	
	Grade 2	1 (3%)	5 (56%)	6 (15%)	
	Grade 3	1 (3%)	2 (22%)	3 (8%)	
Week of final blood sample	Median (range)	5 (1–6)	5 (2–6)	5 (1–6)	0.473

ALC, absolute lymphocyte count; CI, confidence interval; CTCAE, Common Terminology Criteria for Adverse Events; CTV, clinical target volume; EDIC, effective dose to circulating immune cells; ID-TV, integral dose to the target volume; IDVe, integral dose to the segmented vascular tree; RT, radiotherapy; SD, standard deviation. Anatomical grouping refers to the region containing the irradiation target. The photon subgroup reflects an ongoing recruitment stream and is not a randomised comparator. ^1^ Proton versus photon. Continuous variables were compared with Welch’s *t*-test; categorical variables with Fisher’s exact test, extended to the Fisher–Freeman–Halton exact test for variables with more than two categories. The chi-square test is not reported: the minimum expected cell count across the categorical comparisons ranged from 0.69 to 3.00, so the approximation is not valid in this cohort. No correction for multiple comparisons has been applied.

**Table 2:** Flow-contrast MR acquisition parameters.

Parameter	Value
Scanner	3 T MAGNETOM Vida, syngo MR XA20A (Siemens Healthineers, Erlangen, Germany)
Morphological sequence	3D T1 VIBE Dixon (Siemens Healthineers, Erlangen, Germany)
Flow sequence	3D phase-contrast prototype (Advanced Flow Imaging work-in-progress package)
Velocity-encoding directions	3 orthogonal directions (right–left, anterior–posterior, through-plane)
Flow acquisitions per patient	2 (arterial- and venous-optimised)
VENC, head and neck	50 cm/s (arterial); 20 cm/s (venous)
VENC, thorax, abdomen, pelvis	Raised for the descending aorta and inferior vena cava
Respiratory motion control	ReCAR navigator (respiratory-position-driven k-space ordering), thorax and abdomen
Reconstructed images per acquisition	3 phase images + 1 MSUM image + 1 rephased image (no VENC)
Imaging volume	Matched to planning CT coverage; ≈ 20–22 cm craniocaudal
Spatial resolution	1 mm (head), 1.7 mm (body); per-axis range 0.75–2 mm
Added acquisition time	10–20 min for both flow acquisitions
Vessel segmentation	Semi-automated region growing on MSUM in syngo.via, reviewed by a radiation oncologist
Export	Vessel mask to RayStation (RaySearch Laboratories, Stockholm, Sweden) as an organ at risk
Velocity post-processing	Aliasing correction and 3D velocity-field reconstruction in MATLAB R2022a (The MathWorks Inc., Natick, MA, USA)

PC, phase contrast; VENC, velocity encoding; MSUM, magnitude sum; VIBE, volumetric interpolated breath-hold examination.

**Table 3:** Dosimetric metrics by irradiation modality and by lymphopenia status (whole series, *n* = 39).

Grouping	Subgroup	*n*	EDIC (Gy)	IDVe (Gy·L)
Whole series	All patients	39	2.25	1.18 (*n* = 38)
Irradiation modality	Photon RT	9	5.60	3.60 (*n* = 8)
	Proton RT	30	1.25	0.53
Lymphopenia during RT	Yes	9	4.84	3.08 (*n* = 8)
	No	30	1.47	0.67

EDIC, effective dose to circulating immune cells; IDVe, integral dose to vessels; RT, radiotherapy. Values are group means; comparisons by Welch’s *t*-test. IDVe was retrievable in 38 of 39 patients; where the number contributing to a cell differs from the row *n* it is given in parentheses.

**Table 4:** Dosimetric metrics by anatomical target region in the proton cohort.

Target Region	*n*	EDIC (Gy)	IDVe (Gy·L)	ID-TV (Gy·L)
Total	30	1.25	0.53	12.09
Brain ^a,1^	11	0.81	0.13	10.10
Head and neck ^b,2^	12	1.11	0.26	7.80
Thorax–abdomen ^c^	7	2.17	1.63	22.57
Significance		*p* < 0.05 ^a–c^	*p* < 0.05 ^a–c; b–c^	*p* < 0.05 ^b–c^

EDIC, effective dose to circulating immune cells; IDVe, integral dose to vessels; ID-TV, integral dose to target volume. Superscripts denote the groups compared: ^a^, brain; ^b^, head and neck; ^c^, thorax–abdomen. Within-region comparison of start versus end by the paired *t*-test; comparison between regions by Welch’s *t*-test. ^1^ Brain group: 11 patients; the split between focal irradiation and low-dose whole-brain irradiation with a focal boost is to be confirmed by the treating physicians. ^2^ Head-and-neck group: 4 patients with bilateral neck irradiation and 8 patients with unilateral or focal irradiation.

**Table 5:** Lymphocyte counts and kinetics by anatomical target region in the proton cohort.

Target Region	*n*	ALC Start (×10^3^/µL)	ALC End (×10^3^/µL)	*p* (Start vs. End)	Δ ALC (×10^3^/µL)	Lowest ALC (×10^3^/µL)
Total	30	1.92	1.43	<0.001	0.50	1.38
Brain ^a^	11	1.94	1.57	0.018	0.37	1.53
Head and neck ^b^	12	1.86	1.36	0.009	0.49	1.32
Thorax–abdomen ^c^	7	2.02	1.31	0.056	0.71	1.23
Significance between regions					n.s.	n.s.

ALC, absolute lymphocyte count. Superscripts denote the groups compared: ^a^, brain; ^b^, head and neck; ^c^, thorax–abdomen. Within-region comparison of start versus end by the paired *t*-test; comparison between regions by Welch’s *t*-test. n.s., not significant.

**Table 6:** Lymphocyte subpopulations by anatomical target region in the proton cohort.

Target Region	*n*	B Start	B End	*p*	T Start	T End	*p*	NK Start	NK End	*p*
Total	30	3.46	2.21	0.009	23.03	17.15	0.002	4.89	3.57	0.010
Brain	11	2.65	2.37	n.s.	21.92	20.15	n.s.	4.50	4.06	n.s.
Head and neck	12	3.75	2.42	n.s.	23.02	15.03	0.027	5.28	3.02	0.029
Thorax–abdomen	7	4.23	1.60	n.s.	24.80	16.07	n.s.	4.83	3.73	n.s.

Values are percentages of leucocytes, expressed as group means. NK, natural killer. Comparison between start and end of treatment by the paired *t*-test. n.s., not significant.

## Data Availability

The data presented in this study are available on reasonable request from the corresponding author. The data are not publicly available because they contain information that could compromise the privacy of research participants.

## Abbreviations

The following abbreviations are used in this manuscript:

ALC	absolute lymphocyte count
CT	computed tomography
DVH	dose–volume histogram
EDIC	effective dose to circulating immune cells
ID-TV	integral dose to the target volume
IDVe	integral dose to vessels
IMRT	intensity-modulated radiation therapy
MRI	magnetic resonance imaging
MSUM	magnitude sum image
NK	natural killer
OAR	organ at risk
OS	overall survival
PC	phase contrast
PTV	planning target volume
ReCAR	respiratory-position-driven navigator
RIL	radiation-induced lymphopenia
RT	radiotherapy
TPS	treatment-planning system
VENC	velocity encoding
VIBE	volumetric interpolated breath-hold examination
